# Editorial: Machine Learning and Network-Driven Integrative Genomics

**DOI:** 10.3389/fgene.2021.660201

**Published:** 2021-03-11

**Authors:** Mehdi Pirooznia, Shizhong Han, Richard S. Lee

**Affiliations:** ^1^Bioinformatics and Computational Biology Laboratory, National Heart, Lung, and Blood Institute National Institutes of Health, Bethesda, MD, United States; ^2^Department of Psychiatry and Behavioral Sciences, Johns Hopkins School of Medicine, Baltimore, MD, United States; ^3^Lieber Institute for Brain Development, Johns Hopkins Medical, Baltimore, MD, United States

**Keywords:** genomics, integrative analysis, systems biology, neural-network, machine learning

Rapid advances in high-throughput technologies have produced distinct biomedical data sets that can be analyzed using mathematical and statistical models including network science tools to decode interactions among functional molecules in living cells. Availability of data and analysis tools was critical in forming the foundation for complex networks. In the past decade, since the birth of this discipline, a robust conceptual framework known as network biology has emerged. Understanding the dimension and dynamic properties of biological data, including gene-gene and protein-protein interactions, and metabolic networks and pathways, can help elucidate the functional properties of cells, which will eventually assist further in understanding their development and disease dynamics. Machine learning (ML), on the other hand, can handle heterogeneous data in different ways such as naive Bayesian Network data integration, Tree-Based Methods such as Random Forest, and penalized linear models such as LASSO. ML-based omics analyses provide assorted integrative analysis of multiple omics data, by analyzing different omics layers together. The discipline of Network biology is rapidly emerging with most recent applications to personalized medicine. Despite great success, there remain many technical challenges, one of which is how to integrate or transform subject-specific knowledge in order to adapt to deep-learning (DL) algorithms and improve outcomes. Technical hurdles exist in data preprocessing, model selection, parametric function approximation, and model regularization and optimization. This Research Topic addresses these challenges and hurdles with a specific focus on the application of DL algorithms to disease prediction and diagnosis, which has not been adequately explored.

As summarized below, this collection of original research papers presents a significant amount of progress made in the above-mentioned scope of the Research Topic:

CL-PMI identifies pre-miRNA using neural network. In their study, Wang, Ma et al. proposed a pre-miRNA identification algorithm based on a cascaded CNN-LSTM framework, called CL-PMI. They used a convolutional neural network (CNN) and employed long short-term memory (LSTM) to automatically extract features and obtain the sequential and spatial characteristics of pre-miRNAs and capture time characteristics of pre-miRNAs to improve attention mechanisms for long-term dependence modeling. Their method overcomes the dataset imbalance problem and improves the performance of pre-miRNA identification methods.

Inferring Bayesian network using genetic node ordering. In order to study the impact of genetic variations on gene regulatory networks, Wang, Audenaert et al. proposed an alternative method for inferring high-quality Bayesian gene networks. Their method, which is easily scalable to thousands of genes, first constructs a node ordering by conducting pairwise causal inference tests between genes and then allows the user to infer a Bayesian network via a series of independent variable selection problems. In addition to higher sensitivity, this method allows for a unified false discovery rate control across genes and individual edges, and therefore provides a suitable way for tuning the sparsity level of the inferred network.

Identification of genes involved in Fetal Growth Restriction (FGR) by in-depth strategy combining methylomics and transcriptomics analyses. Chabrun et al. performed a rigorous multi-omics approach by combining methylomics and transcriptomics analyses on 36 placenta samples in a case-control study to study pathogenic mechanisms of FGR. Data-mining algorithms were used to combine the analysis of more than 1,200 significantly expressed and/or methylated genes. They used machine learning models to explore the phenotypic subgroups (premature birth, birth weight, and head circumference) associated with FGR allowing for a better description of the FGR pathophysiology as well as key genes involved.

A web server to predict Hepatocellular carcinoma (HCC) (Kaur et al.). This study employed large-scale transcriptomic profiling datasets containing a total of 2,316 HCC and 1,665 non-tumorous tissues obtained from 30 studies. They identified a panel of three genes (FCN3, CLEC1B, and PRC1) as a HCC biomarker using different feature selection techniques. The three-genes-based HCC biomarker identified HCC samples in training/validation datasets with an accuracy between 93 and 98%. Furthermore, the prognostic potential of these genes was evaluated on TCGA-LIHC and GSE14520 cohorts using univariate survival analysis. They also developed a web server HCCpred based on the above study to disseminate their tool to the scientific community.

CRISPR/Cas9 Guide RNA Activity Prediction. In order to accurately predict guide RNA (gRNA) on-target efficacy, Zhang et al. proposed CNN-SVR, a novel hybrid system that combines an improved convolutional neural network (CNN)-based method with support vector regression (SVR). The CNN-SVR system is composed of two major components, a merged CNN as the front-end for extracting gRNA features and an SVR as the back-end for regression and predicting gRNA cleavage efficiency. The authors showed that CNN-SVR can effectively learn deeper features of gRNAs and their corresponding epigenetic features, which outperforms available methods in terms of prediction accuracy, generalization, and robustness.

Developing novel computational methods for the inference of novel biological relations from multi-layered networks (Lee, Zhang et al.). Despite advances in analysis, data mining and knowledge discovery of high-dimensional multi-omics biological data remain a great challenge due to the complexity, heterogeneity, and high-dimensionality inherent in the omics data. Network has been widely used to represent relationships among entities in biological systems. In their review, the authors first discuss the properties of biological heterogeneous multi-layered network (HMLN), then surveyed four categories of state-of-the-art methods, namely matrix factorization, random walk, knowledge graph, and deep learning, and demonstrated their applications to omics data integration and analysis.

Infer the regulatory pathway from mixed observational data. In a new approach Zhong et al. presented a Mixed Directed Acyclic Graph (mDAG) algorithm and R package to infer the regulatory pathway from mixed observational data containing both continuous variables such as gene expression and categorical variables such as phenotypes or single nucleotide polymorphisms. Through extensive simulations and real data analysis, they demonstrated that the mDAG method can identify upstream causal factors and downstream effectors linked to a variable and generate hypotheses for causal direction of regulatory pathways capable of recovering a large sparse DAG with limited sample size.

A Network-based functional omics analysis server (Lee, Lee et al.). Cultivated barley is one of the most produced cereal crops worldwide and an important crop species in plant genetics, because it harbors numerous stress response alleles in its genome that can be exploited for crop engineering. In order to study the functional annotation of its genome, Lee, Lee et al. developed the BarleyNet, a co-functional network of 26,145 barley genes, along with a web server for network-based predictions of biological processes. BarleyNet has three complementary network-based algorithms for prioritizing genes to study genetic components of complex traits such as response to environmental stress: a pathway-centric search for candidate genes of pathways or complex traits; a gene-centric search to infer novel functional concepts for genes; and a context-centric search for novel genes associated with stress response to facilitate understanding of the underlying genetic components of complex traits in barley.

Predicting Autism risk genes via machine leaning approaches (Lin et al.). In order to predict Autism spectrum disorder (ASD) risk genes, the authors employed a machine learning-based approach using features from spatiotemporal gene expression patterns in the human brain, gene-level constraint metrics, and other gene variation features. They performed gene ontology enrichment analysis on these predicted risk genes that not only revealed relevant biological processes to ASD such as neuronal signaling, neurogenesis, and chromatin remodeling, but also highlighted other potential mechanisms that might underlie ASD, such as regulation of RNA alternative splicing and ubiquitination pathway related to protein degradation. They demonstrated that human brain spatiotemporal gene expression patterns and gene-level constraint metrics can help predict ASD risk genes.

Gene regulatory network inference methodologies. In their review (Van den Broeck et al.), the authors described experimental methodologies commonly used to identify regulatory interactions and generate gene regulatory networks (GRNs), which provide a blueprint of transcriptional regulations underlying development and environmental responses, including network topology, network size, and transient binding of transcription factors (TFs) to DNA. Additionally, they reviewed network inference techniques that leverage gene expression data to predict regulatory interactions that can identify new regulatory interactions and drive novel hypotheses. They also highlighted the potential of machine learning approaches to leverage gene expression data to predict phenotypic outputs.

A hybrid Approach for microbiome network inferences. Jiang et al. proposed a general framework, HARMONIES, Hybrid Approach foR MicrobiOme Network Inferences via Exploiting Sparsity, to infer a sparse microbiome network from datasets that are often high-dimensional and suffer from uneven sampling depth, over-dispersion, and zero-inflation. HARMONIES utilizes a zero-inflated negative binomial (ZINB) distribution to model the skewness and excess zeros in the microbiome data as well as incorporate a stochastic process prior to sample-wise normalization. This allows inferring a sparse and stable network by imposing non-trivial regularizations based on the Gaussian graphical model. They showed that HARMONIES can outperform other commonly used methods and discover a novel community of disease-enriched bacteria.

## Author Contributions

MP proposed and edited this Research Topic. SH and RL co-edited this Research Topic. All authors made a substantial, direct and intellectual contribution to this Editorial, and approved it for publication.

## Conflict of Interest

The authors declare that the research was conducted in the absence of any commercial or financial relationships that could be construed as a potential conflict of interest.

